# Geopolymer Concrete with Lightweight Artificial Aggregates

**DOI:** 10.3390/ma15093012

**Published:** 2022-04-21

**Authors:** Katarzyna Kalinowska-Wichrowska, Edyta Pawluczuk, Michał Bołtryk, Adam Nietupski

**Affiliations:** 1 Faculty of Civil Engineering and Environmental Sciences, Bialystok University of Technology, Wiejska 45 E, 15-351 Bialystok, Poland; e.pawluczuk@pb.edu.pl (E.P.); m.boltryk@pb.edu.pl (M.B.); 2 Independent Researcher, 15-161 Bialystok, Poland; adam.nietupski@o2.pl

**Keywords:** geopolymers, artificial lightweight aggregate, fly ash–slag mix, SEM-analysis, lightweight geopolymer concretes

## Abstract

This article presents the physical and mechanical properties of geopolymer concrete with lightweight artificial aggregate. A research experiment where the influence of fly ash–slag mix (FA-S), as part of a pozzolanic additive, on the properties of geopolymers was carried out and the most favorable molar concentration of sodium hydroxide solution was determined. The values of three variables of the examined properties of the geopolymer lightweight concrete (GLC) were adopted: X_1_—the content of the pozzolanic additives with fly ash + flay ash–slag (FA + FA-S) mix: 200, 400 and 600 kg/m^3^; X_2_—the total amount of FA-S in the pozzolanic additives: 0, 50 and 100%; X_3_—the molarity of the activator NaOH: (8, 10 and 12 M). In order to increase the adhesion of the lightweight artificial aggregate to the geopolymer matrix, the impregnation of the NaOH solution was used. Based on the obtained results for the GLC’s compressive strength after 28 days, water absorption, dry and saturated density and thermal conductivity index, it was found that the most favorable parameters were obtained with 400 kg/m^3^ of pozzolanic additives (with 50% FA-S and 50% FA) and 10 NaOH molarity. Changes in the activator’s concentration from 8 to 10 M improved the compressive strength by 54% (for a pozzolana content of 200 kg/m^3^) and by 26% (for a pozzolana content of 600 kg/m^3^). The increase in the content of pozzolanic additives from 200 to 400 kg/m^3^ resulted in a decrease in water absorption from 23% to 18%. The highest conductivity coefficient, equal to 0.463 W/m·K, was determined, where the largest amount of pozzolanic additives and the least lightweight aggregate were added. The structural tests used scanning electron microscopy analysis, and the beneficial effect of impregnating the artificial aggregate with NaOH solution was proved. It resulted in a compact interfacial transition zone (ITZ) between the lightweight aggregate and the geopolymer matrix because of the chemical composition (e.g., silica amount), the silica content and the alkali presoaking process.

## 1. Introduction

With the increasing demand for natural aggregates, the production and use of lightweight waste aggregates with a chemical composition similar to ceramsite [1] in geopolymer composites are becoming more popular. It is a good way to utilize, for example, slag [2] and fly ash [3] and to obtain lightweight composites [4] with good compressive strength results [5]. Due to the fact of their favorable properties and easy availability, artificial aggregates made from clay or swelling slate and fly ash are currently popularly used for cement concretes for both structural and insulating purposes. Due to the presence of several technical problems resulting from the porous nature of lightweight aggregate (LWA), precast applications of the material are preferable and much more common in practice in comparison to monolithic ones [6]. The impregnation of lightweight aggregate (LWA) is an alternative method to its premoistening, which is used to limit the loss of fresh concrete’s workability due to the aggregate’s ability to absorb a great amount of mixing water [6]. The aim of this study was to determine the effectiveness of precoating LWAs with cement paste in modifying the properties of concrete composites.

A large number of silica–aluminous substances and other alkaline melts contained in solid waste are similar to the chemical components required for sintered ceramsite, and the preparation of ceramic pellets can be achieved by the reaction between different solid wastes [7]. A common feature of artificial aggregates is a similar oxide composition. They contain aluminosilicates, which in the presence of a suitable silicate, for example, sodium or potassium silicate, with the addition of a strong base usually of a suitable concentration (NaOH or KOH), can lead to a synthesis reaction and, consequently, the formation of a geopolymer [8]. The issues of using lightweight aggregates in cement and geopolymer concrete have been the subject of research for many years. In order to improve their tightness, light porous aggregates can be impregnated with various substances, e.g., natural or synthetic polymers [9,10], sodium carbonate solution [11], sodium silicate [12], or calcium metasilicate [13]. In addition to the expected sealing effect, the main goal is to provide material for phase change in order to improve the parameters, e.g., freeze–thaw resistance or thermal properties [14]. On the other hand, lightweight aggregate (LWA) precoated with silicate or carbonate solutions is mainly used due to the increase in the LWA’s tightness, which affects the parameters of concretes. Niyazi Ugur Kockal and Turan Ozturan [15] investigated the effect of lightweight ash aggregates on the properties of concrete mixtures, the mechanical properties of concrete and the structure of the interfacial transition zone (ITZ). Yiu Lo et al. [16] conducted research using high-carbon fly ash (HCFA–LWA: high-carbon fly ash–lightweight aggregate) for aggregate production. Microscopic examination of the interphase transition zone showed that the cement grout penetrated the rough surface of the aggregate and improved the quality of the contact zone between the cement grout and HCFA–LWA. The strength of lightweight concrete after 7 days of hardening reached approximately 90% of the 28-day strength.

Since the production of cement concrete is associated with a high level of energy consumption and leads to the emission of large quantities of greenhouse gases, especially carbon dioxide (CO_2_), new, greener materials and technologies are being sought [17]. Ordinary Portland cement and concrete are used globally in the construction industry but have major negative environmental impacts [18,19,20,21]. In a report documented by Andrew [20] from the Centre for International Climate Research Oslo (CICERO), Norway, approximately 4 billion tons of cement were produced in 2016 globally [20]. Nearly 50 billion tons of concrete are produced annually all over the world. The cement industry contributes to approximately 10% of the total greenhouse gas emissions globally and utilizes 1.5 billion gigajoules (GJ) of energy annually. For instance, approximately 0.9 tons of CO_2_ are released in the production of 1 ton of cement [22]. In addition, as the geopolymer raw materials are often waste by-products from other industrial processes (e.g., fly ash and blast furnace slag), their use promotes more sustainable practices in the construction industry both in terms of cost (reduction up to 30%) and greenhouse emissions (reductions up to 80%) [23].

It can also be noted that better sustainability and reduction in CO_2_ emissions in geopolymer production are only possible if the production chain and the design of the mixtures are developed with these results in mind. Sodium silicate, for example, has a high energy and environmental cost (≈0.30 kg CO_2_/kg) when produced traditionally. It is necessary to develop alkali-activated products with alternative activators, produced preferably using residual materials and processes that are not aggressive from an environmental point of view [24].

Hasan Assaedi and Thamer Alomayri et al. [25] researched the mechanical properties of PVA (polyvinyl alcohol) fiber-reinforced geopolymer composites containing different amounts of nanosilica. The suitable content of nanosilica was established as 1.0 to 2.0 wt%. The PVA fiber-reinforced geopolymer nanocomposites containing 1.0 and 2.0 wt% nanosilica improved the compressive strength, flexural strength and impact strength in comparison to the PVA fiber-reinforced geopolymer composite without nanosilica. The optimum content of NS was found to be 2.0 wt% in terms of the maximum compressive strength of the composites, which was 25.0% higher than the control composites. Recently, carbon nanotubes have been incorporated to increase the various properties of concrete, including its rheological, mechanical and durability properties. Carbon nanotubes were also found to improve the ITZ between aggregates and the cement matrix [26,27,28].

Ojha et al. [29] prepared mortar samples containing lightweight geopolymer fly ash sand. The mortars conformed to their respective strength requirements but were lower in comparison to the control mix containing fine natural aggregates. The compressive strength of concrete and mortar mixes containing lightweight geopolymer fly ash sand can be improved if an activator solution of slightly higher concentration and increased post-set heat treatment duration is used for the preparation of lightweight geopolymer fly ash sand. For normal grade concrete (in the range of M20 to M30), geopolymer fly ash sand can be a viable and sustainable solution [29]. Wei and Cheng [30] added sodium salts to the mixture of fly ash and water glass, which decreased all analyzed parameters of concretes. After analyzing the effect of light fine-grained aggregate activated with alkali on the physical and mechanical properties of composites, Hatice Oznur Oz and Hasan Erhan Yucel [31] found an improvement in compressive strength. Zou et al. [32] carried out an experimental investigation on sawdust and metakaolin-based geopolymer and found it to be an effective insulation material for buildings. The samples with a water-to-biomass ratio of up to two were found to exhibit low heat conductivity (0.118–0.125 W/m·K) and were potentially suitable for practical application in building insulations [32].

Previous research on the properties of geopolymer with lightweight artificial aggregates was mainly concerned with improvements in the quality of lightweight aggregates and their influence on the performance of cement concretes. This paper presents the properties of geopolymer concretes with lightweight artificial aggregate with pozzolanic additives with contents of fly ash and a fly ash–slag mix with NaOH. The formation of fly ash from coal combustion is increasingly being curtailed due to the European Union’s decision to end the mining and burning of mine-produced coal by 2050 [33]. A significant part of the generated fly ash is used in the production of cement with pozzolanic additives, e.g., road construction or for self-compacting concretes. For this reason, the aim of this research was to determine the degree of replacement of fly ash in lightweight geopolymer concrete (GLC) with an ash–slag mix and the optimal concentration of the activator used in the form of sodium hydroxide was determined. The effect of earlier impregnation of coarse aggregate with NaOH on GLC’s properties was also examined.

## 2. Materials and Methods

### 2.1. Materials

#### 2.1.1. Fly Ash

Silica fly ash with pozzolanic properties meeting the standard requirements of EN 450-1:2012, Fly ash for concrete—Part 1: Definition, specifications and conformity [34], were used in the tests. Fly ash was obtained from the combustion of hard coal at the Ostrołęka Heat and Power Plant. The chemical composition of fly ash (FA) is presented in Table 1. Chemical composition of FA (fly ash) and FA-S (fly ash–slag mix).

**Figure 1:**
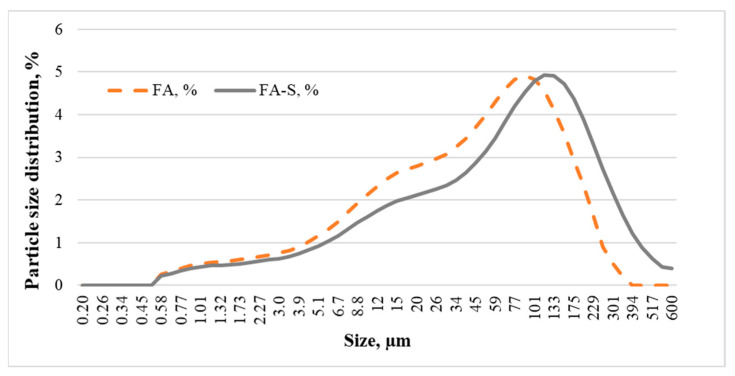
The fly ash and fly ash–slag particle size distributions.

A diagram of the particular size distribution of fly ash is shown in Figure 1.

Fly ash contains 4.37% of unburned carbon (Table 1), which allows it to be classified as category A according to the EN 450-1 [34] standard (LOI ≤ 5%). In Table 2, the physical properties of fly ash and the ash–slag mixture are shown.

#### 2.1.2. Fly Ash–Slag Mix

**Figure 2:**
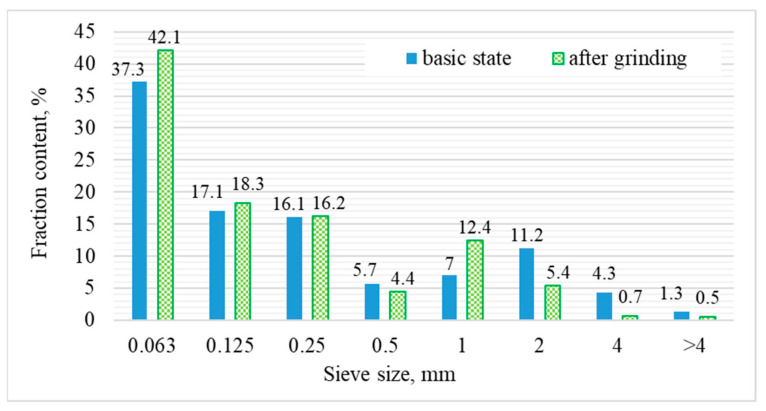
Percentage of the retained FA-S’s fractions in their raw state and after milling.

The fly ash-slag (FA-S) mix was furnace waste in the form of a mixture of fly ash (FA) from electrostatic precipitators and slag from wet storage slags generated at the Kozienice Heat and Power Plant (Poland). The chemical composition of FA-S is shown in Table 1. Table 1 shows that FA-S had a high content of silica, aluminum and lime, and the main components can react with alkalis. Table 2 shows the physical properties of FA-S. It follows that the FA-S mixture was characterized by a higher density than FA, but it had a lower specific surface area. The raw FA-S mixture was characterized by high moisture content, equal to approximately 15%. For this reason, the material was dried to constant weight before testing and then ground in a ball mill for 30 min. The grain size composition of FA-S in its basic state and after grinding is shown in Figure 2.

Drying and milling of FA-S resulted in significant changes in the share of individual fractions. First, there was an increase in the amount of dust fraction observed, from 37.3% to 42.1%, and then an expected decrease in the fraction’s content of >0.5 mm, from 29.5% to 23.4%.

The FA-S was tested for natural radioactivity, and the results are presented in Table 3.

The FA-S meets the requirements for the content of natural radioactive isotopes of potassium K-40, radium Ra-226 and thorium Th-228 in raw materials and materials used in construction and in the control of the content of these isotopes according to the Regulation of the Council of Ministers [35].

### 2.2. Artificial Ash-Lightweight Aggregate

**Figure 3:**
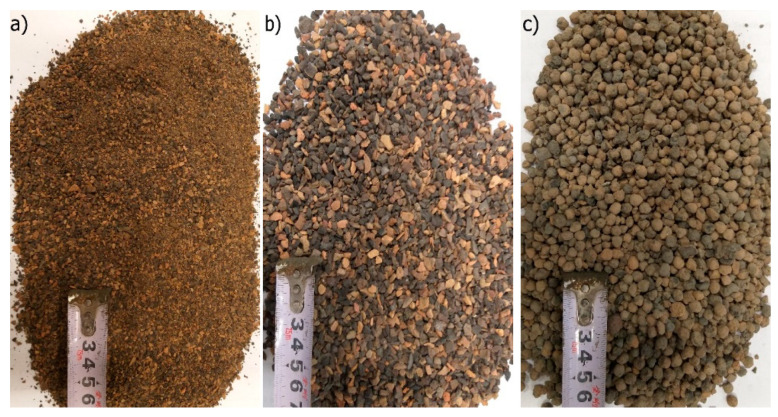
Artificial lightweight aggregate fractions: (**a**) 0–2; (**b**) 1–4; (**c**) 4–8 mm.

Artificial ash-lightweight aggregate, named Certyd [36] and produced in Sowlany near Białystok (Poland), was used in three fractions: 0–2, 1–4 and 4–8 mm, as shown in Figure 3.

The technical characteristics of the individual aggregate fractions are given in Table 4.

Based on the chemical characterization, the contents of aluminum and silica oxides on the surface of the artificial lightweight aggregate were determined as 11.46% and 8.62%, respectively.

**Figure 4:**
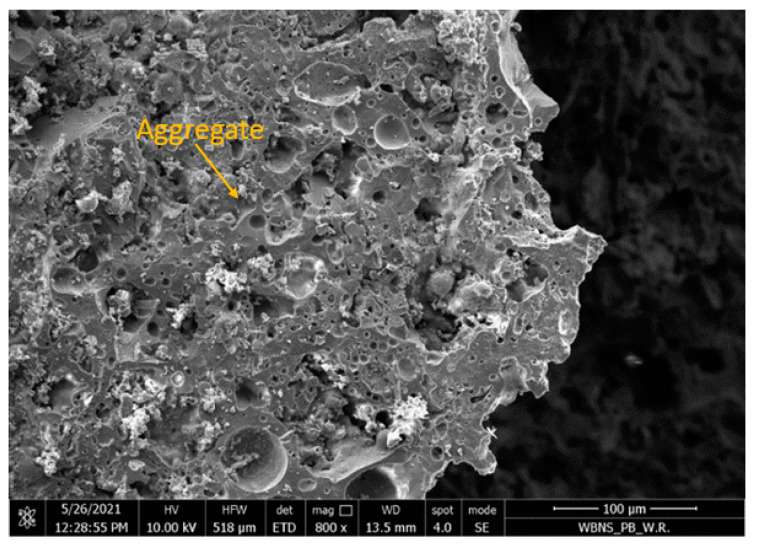
The porous grain structure of the artificial lightweight aggregate (magnified 800×).

**Figure 5:**
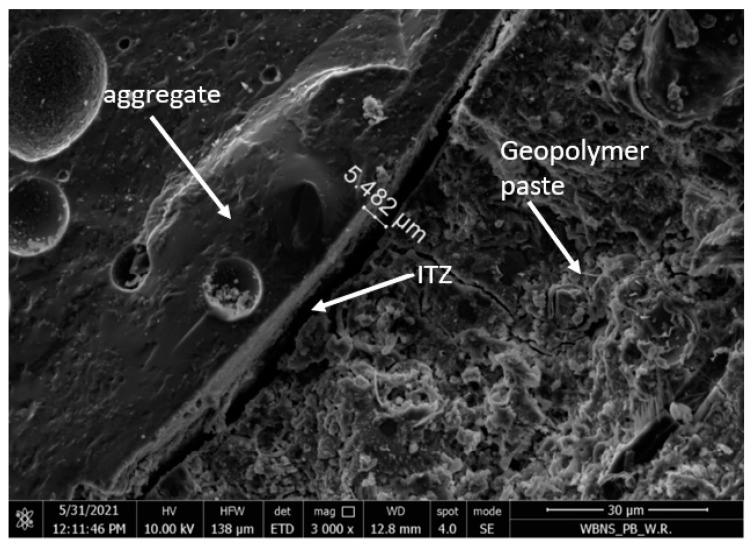
The ITZ between the coarse artificial aggregate and the geopolymer paste (magnified 3000×).

The ash-artificial aggregate of the 4–8 mm fraction was characterized by a porous structure and a developed surface, as shown in Figure 4. Figure 5 shows the leaky interfacial transition zone (ITZ) between the coarse aggregate and the geopolymer paste.

The microstructure of the samples consisted of a homogenous geopolymer gel matrix (Figure 5), dense packing of fly ash and slag particles, along with some minor interstitial porosity and porous aggregate (Figure 4). Silica was reported to be an abundant constituent of geopolymer matrices, and samples with greater silica content showed higher densification and lower porosity levels and were associated with a reduction in pore size and an increase in compressive strength [21,37].

Any material that contains amorphous Si and Al can be used to produce geopolymer concrete. Fly ash and blast furnace slag are the most common waste materials used in geopolymer concrete production [38].

### 2.3. Activator

Three concentrations of sodium hydroxide were used as activators of fly ash and coarse aggregate: 8, 10 and 12 M. These solutions were used for geopolymer mixtures and for the surface impregnation of coarse aggregate (4–8 mm).

## 3. Sample Preparation and Testing Procedures

### 3.1. Purpose of Impregnating Coarse Artificial Aggregate with an Alkaline Solution

Due to the high porosity of the artificial aggregate (Figure 4) and the lack of adhesion of the geopolymer grout to the surface of the coarse aggregate (Figure 5), it was proposed to impregnate the aggregate with the 4–8 mm fraction with an alkali solution.

The tests showed that the surface of the aggregate, as a result of wetting, after 10 s maintained the alkali solution in the amount of approximately 28% of the aggregate’s mass. After the alkali solution hardened on the surface and in the aggregate’s pores, it was tested for water absorption and crushing strength. The test results are presented in Table 5.

The absorbability of impregnated coarse aggregate decreased by more than a half in relation to the nonimpregnated one. The coarse aggregate with the 4–8 mm fraction, which was not impregnated, obtained a crushing index during crushing that was equal to 27.3%. The measure for determining the crushing strength of the aggregate was the crushing index, which for high-class concrete aggregates should be no more than 16%. The crushing indicators are presented in Table 5 and indicate that coarse ash-pore aggregate impregnated with an alkaline solution is suitable for use in high-class concrete, as it obtains a crushing index in the range of 14–15%, and this index decreases with increasing activator concentration.

In further studies, a sodium hydroxide solution was used both for surface impregnation and for the activation of fly ash and the ash–slag mixture (FA-S). For ecological and technological reasons, the addition of water glass, which is commonly used in other studies [39], was abandoned.

Geopolymer mixtures were designed experimentally, assuming their maximum compressive strength and determining their tightness. The process of mixing geopolymer light concrete (GLC) components was carried out in a forced action mixer. The order for dosing the components was as follows: a weighed amount of the coarse aggregate Certyd of 4–8 mm was poured into the mixer; then, a solution of NaOH with a specific concentration was added to the amount according to the experimental plan (NaOH for impregnation of coarse aggregate). After 10 s of mixing, the appropriate amount of fly ash, FA-S and the remaining NaOH solution was poured into the mixer. After 60 s of mixing, the remaining fractions of the Certyd aggregate were poured into the mixer. All ingredients were mixed for another 60 s. In this way, a homogeneous geopolymer blend was obtained.

Then, cubic samples with dimensions of 100 × 100 × 100 mm were formed and compacted in a vibropress station. After shaping, the specimens in the molds were stored for 24 h under a cover in air-dry conditions at a temperature of 20 °C. Then, the samples in the molds were placed in a laboratory dryer and stored for 24 h at a temperature of 65 °C. After maturation, the samples were disassembled and stored for another 26 days in a maintenance basin on grates (above the water’s surface). Twenty-eight days after forming the samples, the physical and mechanical properties of the hardened geopolymer lightweight concrete (GLC) were determined. Tests on the compressive strength were carried out in accordance with the standard EN 12390-3 [40] as well as on water absorption according to PN-88/B-06250 [41], volume density according to EN 12390-7:2019 [42] and thermal conductivity according to EN ISO 6946-10:2017 [43].

Observations of the microstructures of the coarse lightweight aggregate and GLC samples were carried out using a scanning electron microscope (SEM).

### 3.2. Research Plan

The performed laboratory tests were conducted to examine the influence of the presence of the ash–slag mixture, as part of the geopolymer binder, on the properties of the light geopolymer composite and to determine the most favorable molar concentration of the sodium hydroxide solution. The variables proposed to test the properties of geopolymer concrete were:

X₁—The content of pozzolanic additives (FA + FA-S): 200, 400 and 600 kg/m^3^;
$X_{2\;}$—The total amount of FA-S in the pozzolanic additives: 0, 50 and 100%;
$X_{3\;}$—The molarity of the activator NaOH: 8, 10 and 12 M.

In Table 6, the experimental design plan, based on an incomplete plan, with three variables at three levels of each (−1, 0 and 1) and taking into account 14 mix proportions, were shown. Series 15 was made similarly to series 11 for comparison. It contained nonimpregnated aggregate with NaOH.

The amount of activator (NaOH) was prepared at 50% of the number of pozzolanic additives (FA + FA-S), and the amount of NaOH for impregnation was taken as 28% of the weight of 4–8 mm of coarse aggregate. In Table 7, the recipes for the geopolymer blends according to the experimental plan are shown.

Then, tests on the physical and mechanical properties of the geopolymer lightweight concrete were carried out, and the results were analyzed. The test results were statistically analyzed in order to determine an approximating function describing the influence of tested variables on selected properties of geopolymer light concrete. The analyses included the analysis of variance, calculation of the regression coefficients and assessment of the regression coefficients’ significance. The function describing the changes in the physical and mechanical properties of the GLC adopted the form of a second-degree polynomial (1):

(1) $$y=b_{0}+b_{1}\cdot x_{1}+b_{2}\cdot x_{2}+b_{3}\cdot x_{3}+b_{4}\cdot x_{1}x_{2}+b_{5}\cdot x_{1}x_{3}+b_{6}\cdot x_{2}x_{3}+b_{7}\cdot x_{1}^{2}+b_{8}\cdot x_{2}^{2}+b_{9}\cdot x_{3}^{2}$$

where *y* is the dependent variable;$\;x_{1},{\;x}_{2}{\;\mathrm{and}\;x}_{3\;}$ are the independent variables; $b_{i\;\left(i=1,\ldots ,9\right)}$ are the coefficients;$\;b_{0}$ is the intercept of the equation.

where:
*y*—dependent variable;
$x_{1},{\;x}_{2},{\;x}_{3\;}$—independent variables;
$b_{i\;\left(i=1,\ldots ,9\right)}$—coefficients;
$b_{0\;}$—intercept of the equation.

Calculations were performed according to [44] using Statistica version 13.

## 4. Tests Results and Analysis

### 4.1. The Compressive Strength after 28 Days

The average results of the compressive strength tests after 28 days of GLC, water absorption and apparent density are given in Table 8. Each average result was obtained from five measurements.

In Table 9, the regression equations describing the changes in the characteristics of lightweight geopolymer concrete, depending on the variables and taking into account only statistically significant coefficients of the equation (with α = 0.05), are shown.

The statistical analysis showed that all analyzed variables had a statistically significant impact on the compressive strength of GLC at the significance level α = 0.05.

**Figure 6:**
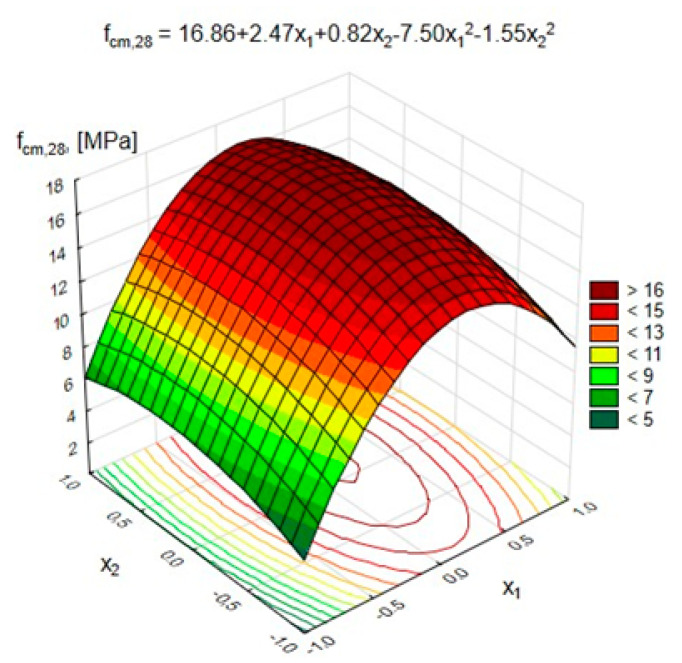
Compressive strength of the GLC after 28 days depending on x_1_ and x_2_ at x_3_ = 0 (10 M).

**Figure 7:**
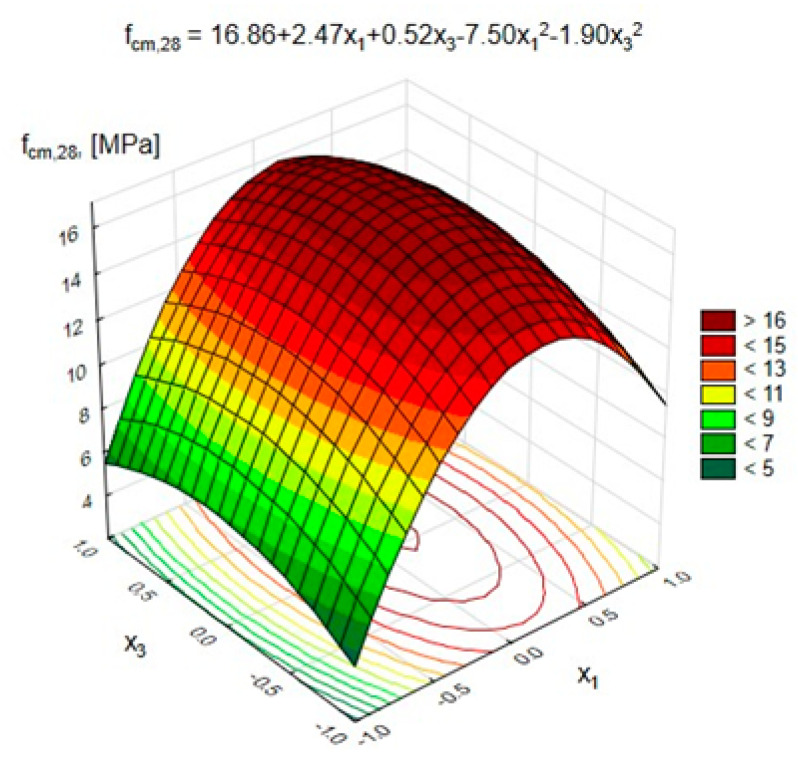
Compressive strength of the GLC after 28 days depending on x_1_ and x_3_ with x_2_ = 0 (50%).

Figure 6 and Figure 7 show the changes in the GLC’s compressive strength after 28 days, depending on the variables x_1_ and x_2_, respectively (at x_3_ = 0, (10 M)), and x_1_ and x_3_ (at x_2_ = 0, (50%)).

Figure 6 shows that the increase in the number of pozzolanic additives from 200 (x_1_ = −1) to 400 kg/m^3^ (x_1_ = 0) resulted in a 1.5–2 times (depending on the content of FA-S) increase in the GLC’s compressive strength after 28 days. On the other hand, a further increase to 600 kg/m^3^ (x_1_ = 1) caused a negative effect in the form of a decrease in strength by, on average, approximately 30%. This was probably due to the excessively high content of fine particles in the geopolymer composite, which primarily increased the water demand of the binder. It also caused difficulties in the proper mixing of ingredients and compaction of the geopolymer blend. On the other hand, increasing the content of FA-S in the geopolymer binder from 0% to 50% resulted in a gradual increase in compressive strength by 52% and 25%, respectively, with the total content of pozzolanic additives equal to 200 and 600 kg/m^3^. When a larger amount of FA-S was used, up to 100% of the weight of additives, an approximately 10% decrease in the GLC’s compressive strength was noted. Thus, the presence of FA-S in the geopolymer binder performed better with a lower total amount of additives to the GLC.

Figure 7 shows that increasing the activator concentration from 8 to 10 M caused an improvement in the compressive strength by 54% (for a pozzolana content of 200 kg/m^3^) and by 26% (for content of 600 kg/m^3^), respectively.

When using the activator with the highest considered concentration, equal to 12 M, a decrease in compressive strength compared to 10 M by approximately 20% (with x_1_ = 200 kg/m^3^) and by 12% (with x_1_ = 600 kg/m^3^) was noted. This means that a high concentration of NaOH causes problems with the workability of the geopolymer blend. It should be considered possible to increase the consumption of the activator in relation to the geopolymer binder, which increases the production costs of lightweight concrete. Thus, from the point of view of compressive strength after 28 days, the most advantageous application is the use of a geopolymer binder in the amount of 400 kg/m^3^ of lightweight concrete at an FA-S content of 50%. However, the optimal activator concentration in the considered experiment was 10 M. In addition, when comparing the compressive strength of series 11, containing previously impregnated aggregate, and series 15 (control), with nonimpregnated aggregate, it should be noted that impregnation of the aggregate increased the compressive strength of the concrete by almost twice. This was due to the improvement in its tightness and strength, which is confirmed by the results presented in Table 5. During the sample loading process in the press, it was noticed that the GLC behaved differently under load than ordinary cement concrete. The first signs of damage in the GLC were observed at approximately 80–90% of the maximum load. Cracks in the GLC structure usually occur through the aggregate and the geopolymer matrix, which proves the equivalent strength parameters of the artificial lightweight aggregate and the geopolymer matrix. The mixture of fly ash and blast furnace slag improved the pore structure and mechanical strength of the geopolymers compared to the exclusive use of fly ash; this mixture also increased the binder’s resistance to the negative effects of acids, sulfates and seawater. Geopolymer utilizes the polycondensation of silica, alumina and high alkali content to attain compressive strength [38,45].

The main reaction products of alkali-activated fly ash were Si and Al; a N–A–S–H gel (Na_2_OAl_2_O_3_-SiO_2_-H_2_O) with a three-dimensional framework of SiO_4_ and AlO_4_ was tetrahedrally linked through shared O atoms. According to chemical composition analysis results for the fly ash used, as shown in Table 1, the total amount of Si and Al in the fly ash used was 84%. Thus, this geopolymer is expected to form a sodium aluminosilicate hydrate (N–A–S–H) gel after the geopolymerization process. The reaction between fly ash and the alkali activator solution forms sodium aluminosilicate hydrates. This hydration product, along with the aluminosilicate structure in the fly ash samples, contributed to increasing the strength [37].

### 4.2. Water Absorption of Lightweight Geopolymer Concretes

The statistical analysis showed that all of the analyzed variables had a statistically significant impact on the absorbability of lightweight geopolymer concrete at the significance level α = 0.05.

**Figure 8:**
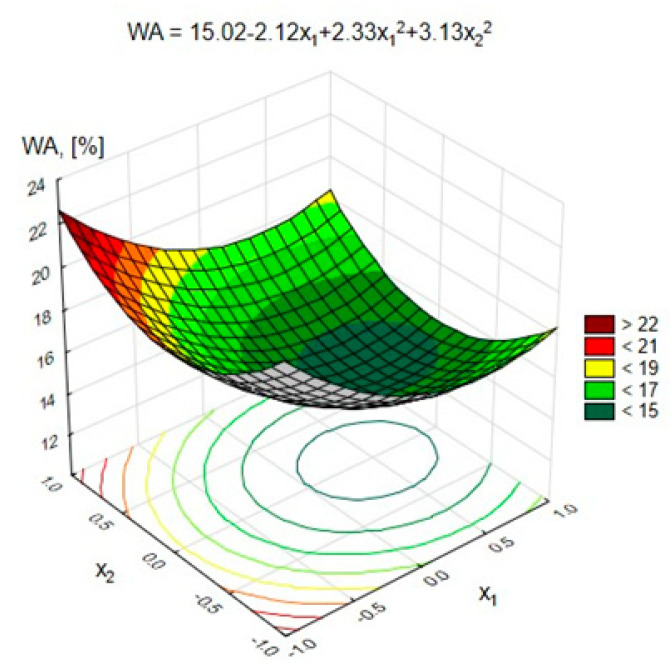
Changes in the water absorption of GLC depending on x_1_ and x_2_ at x_3_ = 0 (10 M).

**Figure 9:**
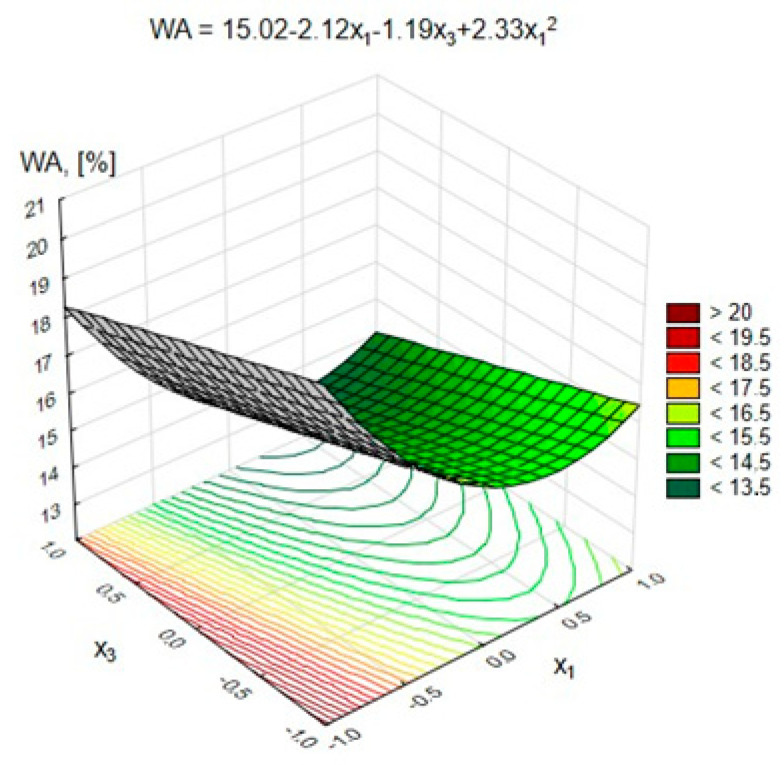
Changes in the water absorption of GLC depending on x_1_ and x_3_ at x_2_ = 0 (50%).

Figure 8 and Figure 9 show the changes in the GLC’s water absorption depending on the variables x_1_ and x_2_, respectively (at x_3_ = 0, (10 M)), and x_1_ and x_3_ (at x_2_ = 0, (50%)).

Figure 8 shows that an increase in the content of pozzolanic additives from 200 to 400 kg/m^3^ resulted in a decrease in water absorption from 23% to 18%. On the other hand, increasing their amount in the geopolymer concrete to 600 kg/m^3^ resulted in a slight increase in water absorption.

A similar relationship was observed in the case of the FA-S mixture. Increasing the amount of FA-S from 0% to 50% of the weight of the pozzolanic additives had a beneficial effect in the form of lowering water absorption from approximately 18% to 15%. A further increase in FA-S resulted in a renewed increase in the absorbability of light geopolymer concrete by approximately 3%. There was also a slight, gradual decrease in water absorption by approximately 2%, with an increasing activator concentration from 8 to 12 M (Figure 9). The presented dependencies show that the concentration of the NaOH solution had the least influence on the GLC’s absorbability. The key factor determining the absorbability of lightweight concrete was the content of pozzolanic additives in the composite and the content of the FA-S mixture in them. The lowest water absorption was achieved for series 14, with the content of pozzolanic additives equal to 400 kg/m^3^ (at 200 kg/m^3^ FA-S) and at a concentration of activator equal to 12 M. When comparing the water absorption of concrete of series 11 to that of series 15 (with the nonimpregnated aggregate), it should be noted that impregnation of coarse aggregate reduced the water absorption from 28.3% to 18.5%.

### 4.3. Apparent Density of Light Geopolymer Concretes

**Figure 10:**
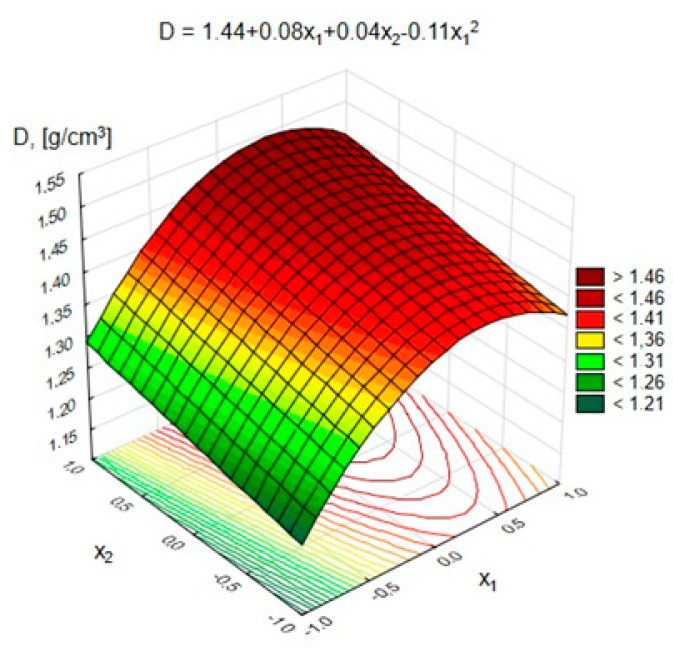
Changes in the volume density of the GLC depending on x_1_ and x_2_.

The statistical analysis of the research results showed that the changes in the GLC’s density were significantly influenced by the variables: x_1_—the total content of pozzolanic additives; x_2_—the content of FA-S. The concentration of the activator (x_3_) was determined to be statistically insignificant in this case. Figure 10 shows the changes in the density of the GLC depending on the coded variables x_1_ and x_2_.

Figure 10 shows that with the increase in the content of pozzolanic additives from 200 to 400 kg/m^3^ in lightweight concrete, the GLC’s volume density increased by an average of approximately 15%. When increasing the number of pozzolanic additives to 600 kg/m^3^, a slight decrease in density was noted, which is synonymous with an increase in water absorption in this range and a deterioration in the compressive strength. The increase in the amount of FA-S in the binder caused an increase in the concrete’s density by approximately 6–7%, which resulted from the higher density of FA-S in comparison to FA. The activator concentrations did not have a significant effect on the density of the concrete and, therefore, were not considered here.

### 4.4. Tests on the Heat Conductivity Coefficient λ (W/mK)

**Figure 11:**
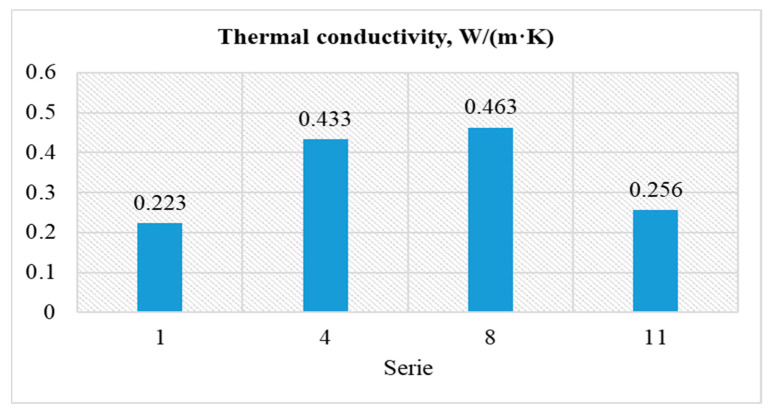
Values for the thermal conductivity coefficient “λ”.

The study of the heat conductivity coefficient was established for selected series with different contents of pozzolanic additives and activator concentrations. The tests were carried out on three samples with dimensions of 300 × 300 × 50 mm for series 1, 4, 8 and 11. The average results of the λ factor are presented in Figure 11.

The lowest value for the thermal conductivity coefficient “λ” of 0.223 W/m·K was obtained for series 1, which contained the least pozzolanic additives, and thus was the most lightweight aggregate. The highest conductivity coefficient, equal to 0.463 W/m·K, was determined for series 8, containing the largest amount of pozzolanic additives and was the least lightweight aggregate. The obtained test results indicate that the amount of Certyd artificial aggregate in 1 m^3^ of geopolymer mix is of key importance in the case of the tested feature. As low as it was in series 1, the lambda value was obtained thanks to the porosity of the artificial aggregate and the high porosity of the geopolymer matrix. The effect of the NaOH activator concentration on the λ factor can be observed by comparing the results of series 4 and 8, where the lambda increased by 0.03 W/m·K, i.e., by 6.9%, with the increase of the activator concentration from 8 to 12 M.

## 5. SEM Observations of Microstructure of the GLC

**Figure 12:**
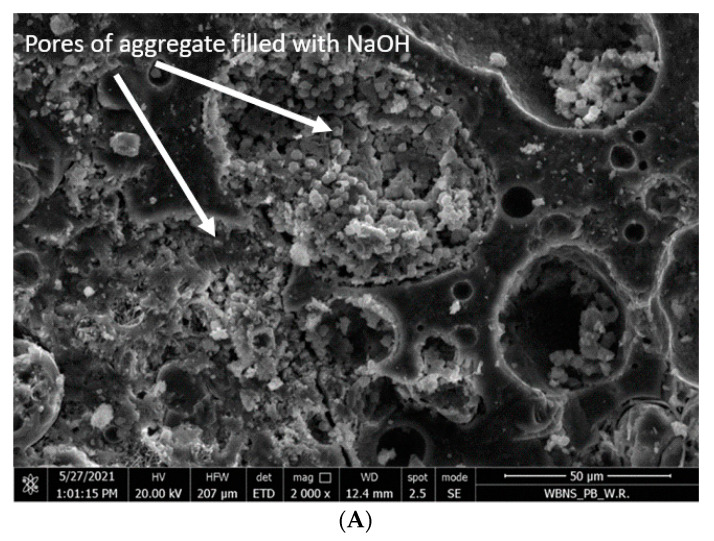

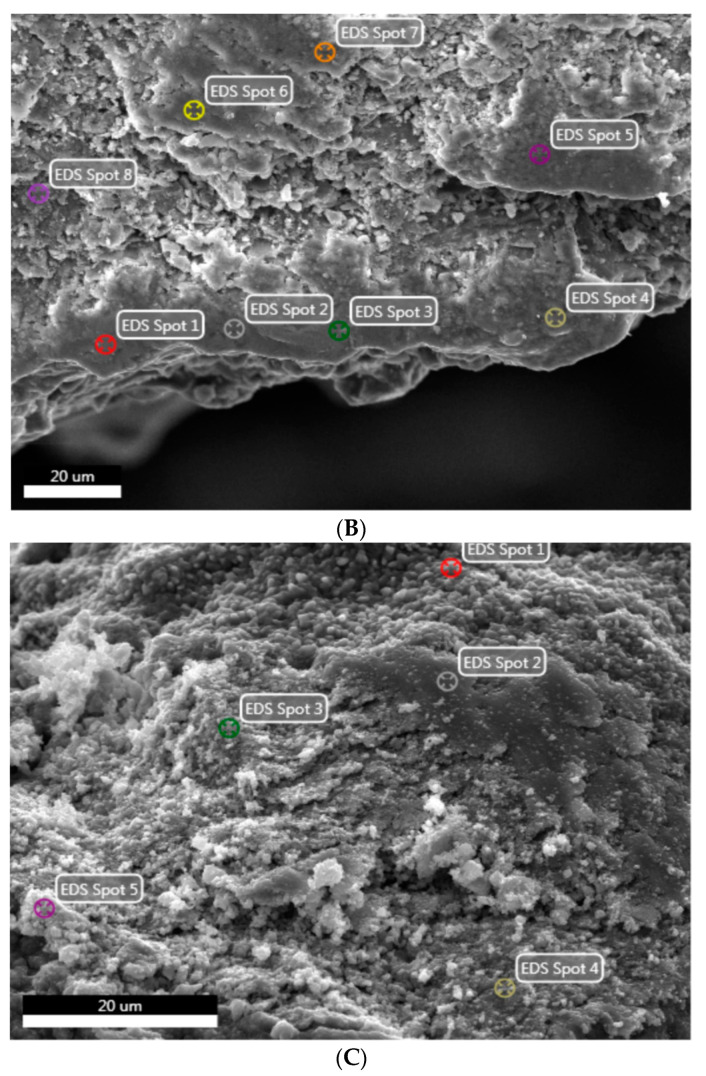
(**A**) Light aggregate pores filled with NaOH solution (magnification 2000×). (**B**) The surface of LWA in geopolymer matrix separated for EDS spots. (**C**) The EDS spots marked inside LWA.

Scanning photos of the microstructure of the examined geopolymer concrete on artificial aggregate were analyzed using a scanning electron microscope Quanta 250 FEG, FEI, Hillsboro, OR, USA. Figure 12A shows the structure of the geopolymer concrete with a coarse aggregate with the 4–8 mm fraction, impregnated with NaOH solution at a concentration of 12 M.

Figure 12A shows larger pores in the coarse aggregate partially filled with NaOH solution. This proves that the impregnation with NaOH solution resulted in a mechanical and chemical connection of the geopolymer mortar with the texture of coarse aggregate [38]. By using the EDS technique, the chemical components on the surface and inside the lightweight aggregate (LWA) in the geopolymer matrix were carried out. The results are shown in Figure 12B and Table 10 and also in Figure 12C and Table 11.

The chemical components detected in the Figure 12B and in Table 10 (Al, Na, O, Si) show that sodium silicates formed on the surface of the lightweight aggregate as a result of the reaction of aluminum oxides on its surface with NaOH, which is also described in [37]. Different areas were found inside the LWA aggregate (Figure 12B and Table 11).

As the results of chemical components show (Figure 12C and Table 11), inside the LWA Na and O are detected, which confirm the presence of NaOH in there.

**Figure 13:**
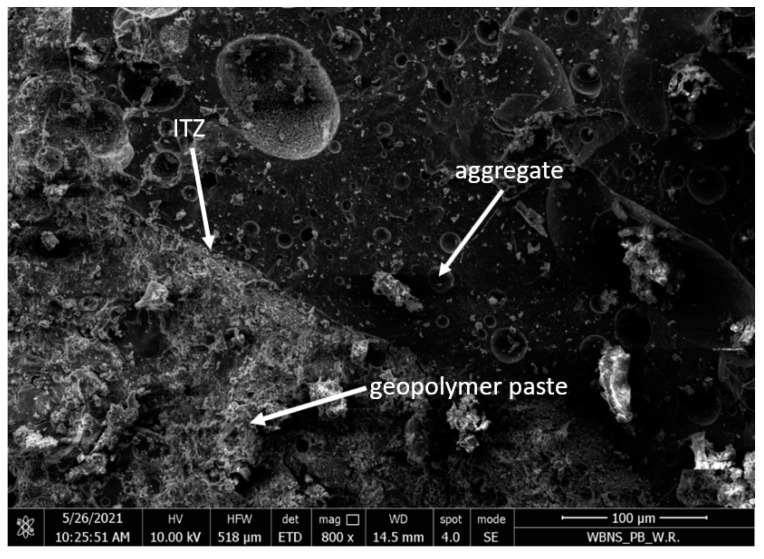
ITZ between impregnated lightweight aggregate and geopolymeric paste (magnification 800×).

**Figure 14:**
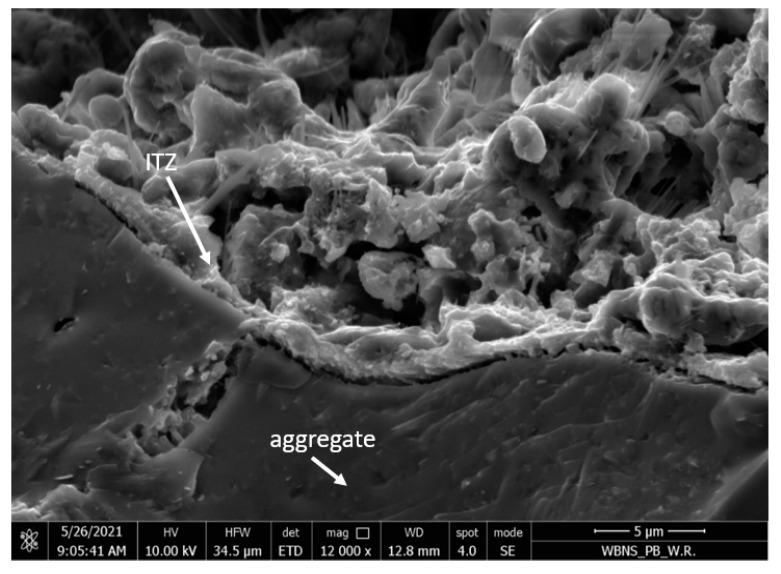
ITZ between the impregnated aggregate and the geopolymeric paste (magnification 12,000×).

Among other things, for these reasons, a tighter structure of the contact layer between the geopolymer mortar and the surface of the artificial aggregate was obtained (Figure 13 and Figure 14), in addition to better strength parameters [37].

## 6. Summary

- Due to the high content of aluminosilicates in FA, FA-S and the artificial aggregate, it was possible to use a NaOH solution as an alkali activator, which resulted in approximately 15–20 MPa of compressive strength for the GLC;
- In order to improve the quality of the ITZ between the artificial aggregate and the geopolymer matrix, the impregnation by NaOH solution of coarse aggregate is also recommended;
- The optimal content of FA-S should be assumed per 50% of approximately the mass of FA for the best results in compressive strength after 28 days;
- From the statistical analysis based on the compressive strength after 28 days, water absorption and density tests on GLC, the optimal composition of lightweight geopolymer concrete was determined to be: FA 200 kg/m^3^, FA-S 200 kg/m^3^; 10 M NaOH solution; an artificial aggregate fraction of 0–2 mm for 222 kg/m^3^; 1–4 mm for 223 kg/m^3^ and 4–8 mm for 445 kg/m^3^; for surface impregnation of the coarse aggregate, a fraction of 4–8 mm with 125 L of NaOH;
- As per the optimization tests on the influence of the percentage of FA-S in relation to the silicate fly ash, the content of NaOH solution and its molar concentration showed that their best composition could be obtained, affecting GLC with compressive strengths over 16 MPa. If there is a need to obtain higher compressive strength, two activators, sodium water glass and sodium hydroxide, should be used;
- The composites from the GLC could be used for insulating materials, where their high strength is not required.

## Figures and Tables

**Table 1:** The chemical composition of fly ash (FA) and ash–slag (FA-S).

Chemical Composition	FA, %	FA-S, %
SiO₂	54.6	42.3
Fe_x_O_y_	4.97	3.78
Al₂O₃	25.3	17.1
Mn₃O₄	0.06	0.07
TiO₂	1.07	0.77
CaO	2.14	11.30
MgO	1.8	5.25
SO₃	0.37	0.13
P₂O₅	0.55	0.45
Na₂O	0.84	0.61
K₂O	2.8	1.77
BaO	0.15	0.09
SrO	0.07	0.06
LOI	4.37	15.72
Summary	99.09	99.40
Moisture	0.12	0.5
free CaO	<0.03	<0.03
Reactive SiO_2_	42.36	24.8
Reactive CaO	1.84	<0.01

**Table 2:** Physical properties of the fly ash (FA) and ash–slag (FA-S) mixture.

Properties	FA	FA-S
Specific density, g/cm^3^	2.13 (0.02)	2.35 (0.03)
Bulk density, g/cm^3^	1.15 (0.03)	1.22 (0.03)
Specific surface area according to Blaine’s method, cm^2^/g	3700 (81.9)	2450 (64.3)

**Table 3:** The results of the FA-S natural radioactivity test.

Activity Index $f_{1}$	Activity Index $f_{2}$, Bq/kg	Exposure Dose Strength MD, μGy/h
0.76	77.30	0.100

Where $f_{1}$ is the activity index determining the content of natural radioactive isotopes, $f_{2}$ is the activity indicator determining the radium content Ra-226.

**Table 4:** Artificial lightweight aggregate fractions of 0–2, 1–4 and 4–8 mm [36].

Properties	Unit	Standard	Fraction, mm		
			0–2	1–4	4–8
Bulk density	g/cm^3^	EN 1097-3	0.90 ± 10%	0.62 ± 10%	0.70 ± 10%
Specific density	g/cm^3^	EN 1097-6	-	-	1.30 ± 0.15
Water absorption after 24 h	%	EN 1097-6	-	-	20
Crush resistance	N/mm^2^	EN 13055-1 part A	-	-	>6
Frost resistance	%	EN 13055-1 part C	-	-	1.0
Chlorides content	%	EN 1744-1	0.00		
Acid-soluble sulfate content	%	EN 1744-1	0.25		
Total sulfur content converted to S.	%	EN 1744-1	0.32		
The content of organic impurities		EN 1744-1	lighter color than the reference		
Alkaline reactivity (fast method)		PN-B 06714/46	0.00		
Radioactivity	Bq/kg	ITB Instruction No. 455/2010	*f*_1_ ≤ 1.2 *f*_2_ ≤ 240		
Thermal conductivity λ	W/m·K	EN 12667	0.18 (dry)	0.16 (dry)	0.14 (dry)

**Table 5:** The properties of the lightweight artificial aggregate with the 4–8 mm fraction.

Properties	Unit	Lightweight Aggregate 4–8 mm after Impregnation			Lightweight Aggregate 4–8 mm Not Impregnated
		8 M	10 M	12 M	
Water absorption	%	10.2	9.8	9.4	23.5
Crushing indicator	%	15.2	14.5	14.0	27.3

**Table 6:** The design of the experiment with real and coded variables.

Series	Real Value			Coded Value		
	X_1_, kg/m^3^	X_2_, %	X_3_, M	x_1_	x_2_	x_3_
1	200	0	8	−1	−1	−1
2	600	0	8	1	−1	−1
3	200	100	8	−1	1	−1
4	600	100	8	1	1	−1
5	200	0	12	−1	−1	1
6	600	0	12	1	−1	1
7	200	100	12	−1	1	1
8	600	100	12	1	1	1
9	200	50	10	−1	0	0
10	600	50	10	1	0	0
11	400	0	10	0	−1	0
12	400	100	10	0	1	0
13	400	50	8	0	0	−1
14	400	50	12	0	0	1
15	400	0	10	Aggregate 4–8 mm not impregnated		

**Table 7:** The geopolymer mixtures per 1 m^3^.

Series	FA	FA-S	NaOH	Molarity of NaOH	NaOH Used for Impregnation	Lightweight Aggregate		
						0–2 mm	1–4 mm	4–8 mm
	kg	kg	dm^3^	M	dm^3^	kg	kg	kg
1	200	0	100	8	150.9	269.5	269.5	539.0
2	600	0	300	8	88.7	158.4	158.4	316.8
3	0	200	100	8	150.9	269.5	269.5	539.0
4	0	600	300	8	88.7	158.4	158.4	316.8
5	200	0	100	12	150.9	269.5	269.5	539.0
6	600	0	300	12	88.7	158.4	158.4	316.8
7	0	200	100	12	150.9	269.5	269.5	539.0
8	0	600	300	12	88.7	158.4	158.4	316.8
9	100	100	100	10	150.9	269.5	269.5	539.0
10	300	300	300	10	88.7	158.4	158.4	316.8
11	400	0	200	10	124.7	222.6	222.6	445.2
12	0	400	200	10	124.7	222.6	222.6	445.2
13	200	200	200	8	124.7	222.6	222.6	445.2
14	200	200	200	12	124.7	222.6	222.6	445.2
15	400	0	200	10	0	222.6	222.6	445.2

**Table 8:** The average test results for compressive strength after 28 days, water absorption and the apparent density of the GLC.

Series	X_1_	X_2_	X_3_	$f_{cm,28}$, MPa	*WA*, %	*D*, g/cm^3^
1	200	0	8	2.54	25.6	1.21
2	600	0	8	6.85	18.6	1.47
3	200	100	8	4.47	23.1	1.28
4	600	100	8	7.68	19.1	1.54
5	200	0	12	3.10	20.4	1.35
6	600	0	12	8.14	17.8	1.33
7	200	100	12	3.86	20.4	1.38
8	600	100	12	10.66	17.1	1.54
9	200	50	10	6.69	19.5	1.28
10	600	50	10	12.02	15.2	1.38
11	400	0	10	14.24	18.5	1.47
12	400	100	10	16.38	17.8	1.52
13	400	50	8	14.45	15.4	1.47
14	400	50	12	15.47	14.2	1.41
15	400	0	10	7.23	28.3	1.35

**Table 9:** Regression equations.

Properties	Equation	$R^{2}$
Compressive strength, $f_{cm,28}$, MPa	$=16.86+2.47\cdot x_{1}+0.82\cdot x_{2}+0.52\cdot x_{3}-7.50\cdot x_{1}^{2}-1.55\cdot x_{2}^{2}-1.9\cdot x_{3}^{2}$	0.98
Water absorption, *WA*, %	$=15.02-2.12\cdot x_{1}-1.19\cdot x_{3}+2.33\cdot x_{1}^{2}+3.13\cdot x_{2}^{2}$	0.97
Volume density, *D*, g/cm^3^	$=1.44+0.08\cdot x_{1}+0.04\cdot x_{2}-0.11\cdot x_{1}^{2}$	0.96

**Table 10:** The chemical components detected on the surface of LWA impregnated with NaOH.

Element	Weight %	Atomic %
O	44.8	57.2
Na	15.9	14.1
Al	7.6	5.8
Si	31.7	23.0

**Table 11:** The chemical components detected on the surface of LWA in geopolymer matrix.

Element	Weight %	Atomic %
C	14.1	21.0
O	36.5	40.7
Na	48.5	37.7
Si	0.9	0.5

## Data Availability

Not applicable.
